# Validation of the Zung self-rating depression scale (SDS) in older adults

**DOI:** 10.1080/02813432.2019.1639923

**Published:** 2019-07-09

**Authors:** Jari Jokelainen, Markku Timonen, Sirkka Keinänen-Kiukaanniemi, Pirjo Härkönen, Heidi Jurvelin, Kadri Suija

**Affiliations:** a Center for Life Course Health Research, University of Oulu, Oulu, Finland;; b Unit of General Practice, Oulu University Hospital, Oulu, Finland;; c Health Center of Oulu, Oulu, Finland;; d Department of Family Medicine, University of Tartu, Tartu, Estonia

**Keywords:** BDI-21, Diagnostic methods, elderly, major depression, statistics

## Abstract

**Objective:** The main objective of this study was to investigate the psychometric properties of the Zung Self-Rating Depression Scale (SDS) and evaluate screening parameters capability of the SDS with the Beck Depression Inventory (BDI-21) among the elderly population.

Design: A population-based study

**Setting:** Community

**Subjects:** 520 adults, aged 72–73 years, living in the city of Oulu, Finland.

Main outcome measures: The screening parameters of the SDS questions and BDI-21 for detecting severity of depression. The Mini Neuropsychiatric Interview for diagnosing major depression.

**Results:** The optimal cut-off point for the SDS was 39. The sensitivity and specificity parameters for this cut-off point were 79.2% (95% CI 57.8–92.9) and 72.2% (95% CI 67.9–76.1), respectively. Positive and negative predictive values were 12.5% (95% CI 7.7–18.8) and 98.6% (95% CI 96.7–99.5), respectively. Moreover, there was no statistically significant difference in diagnostic accuracy indices of the cut-off points 39 and 40. In a receiver operating characteristic analysis, the area under the curve was 0.85 (95%CI 0.77–0.92) for the SDS total score and 0.89 (95% CI 0.83-0.96) for the BDI-21 (*p* = 0.137).

**Conclusion:** Using the traditional cut-off point, the SDS was convenient for identifying clinically meaningful depressive symptoms in an elderly Finnish population when compared with the BDI-21 which is one of the most commonly used depression screening scales. The sensitivity and specificity of these two screening tools are comparable.

Based on our study, the SDS is convenient for identifying clinically meaningful depressive symptoms among older adults at the community level.

**Key points**
- The widely used Zung Self-Rating Depression Scale (SDS) has not previously been validated among elderly people at the community level.
- The sensitivity and specificity of SDS (cut-off point 39) were 79.2% and 72.2%.
- The positive and negative predictive values for SDS were 12.5% and 98.6%.
- SDS is convenient for identifying major depression in an elderly population and regarding sensitivity and specificity comparable to BDI-21.

## Introduction

Diagnosing depression can be a challenging process, especially if the subject is elderly and has co-morbid medical conditions mimicking symptoms of depression [1]. Diagnostic criteria for major depression include sadness or low mood, loss of interest in or pleasure in daily activities, and reduced energy nearly every day for more than two weeks; together with having some of the following specific symptoms: reduced self-esteem, feelings of guilt, disturbed sleep, change in appetite, problems in concentration, change in activity, ideation of self-harm; and impaired function [2,3]. The criteria applied to the general population are also used for diagnosis in elderly people. However, studies have shown that under-recognition of depression is very prevalent in the elderly, especially at the community level [4,5].

Despite this, depression is a common problem in older age, with 11–29% of subjects aged over 65 years exhibiting depressive symptoms [6–8]. Therefore, diagnosis of depression in elderly patients with co-existing medical illnesses is a challenge for the physician and there is an evident need for instruments that can assist in the diagnostic process.

Depression diagnosis is a clinical process, where results from structured interviews ca also be utilized [9]. Several self-rating scales are also available, some of the self-rating scales have also been, at least partially, validated in elderly populations [10,11]. The most commonly used self-rating questionnaires for depression in the elderly are the Beck Depression Inventory (BDI), the Geriatric Depression Scale (GDS), the Zung Self-Rating Depression Scale (SDS) and Patient Health Questinnaire (PHQ) [10,12–16]. With its good reliability and validity, the BDI is probably the most frequently used questionnaires for the assessment of depression in clinical practice [17,18]. The GDS was specially designed to measure depression in geriatric patients [14]. The SDS is a short self-rated scale that assesses the psychological and somatic symptoms of depression. It has been widely used in various age groups for both screening purposes and for measuring depression [17,19–23]. However, most previous studies that have validated the SDS were conducted among patients attending clinical visits. We are aware of only one study conducted among a general population [24] and one report summarizing studies using the SDS in elderly populations [25]. Thus, there exists a need to evaluate the psychometric properties of this widely used questionnaire among elderly people at the community level.

The main objective of this study was to investigate the psychometric properties of the SDS using the Diagnostic and Statistical Manual of Mental Disorders (DSM-IV) diagnosis of depression as a gold standard in older adults at the community level; the second objective was to compare the screening parameters of the SDS with those of the BDI among the elderly population.

## Material and methods

This prospective population-based study was conducted between 1990 and 2008. We invited all persons (*n* = 1008) born in 1935 and living on 1 October 1990 in the city of Oulu, Northern Finland, to participate in the study. This study is based on the second follow-up, which took place in 2007–2008. Altogether 667 persons who were alive were asked to attend on the second follow-up and 520 of them completed the study, without any excluding criteria.

The study protocol was approved by the Ethics Committee of the Faculty of Medicine, University of Oulu.

A detailed description of the enrolment procedure and the objectives of the study have been described previously [26–28]. Briefly, all participants gave informed consent. Data were collected via questionnaires, interviews, and clinical examinations. Standardized questionnaires were used to obtain self-reported data on sex, education, marital status, and medical conditions diagnosed by a physician. All questionnaires were filled by the participants at home or the study centre. The returning of questionnaires was supervised by a research assistant, who also instructed to fill questionnaires if needed.

For assessment of the patient’s subjective view of his/her depressive symptoms, we used the SDS and the 21-item version of the Beck Depression Inventory (BDI-21) [15,16]. The SDS contains 20 items and its design was based on the diagnostic criteria for depression. Subjects rate each item with regard to how they have felt during the past several days using a 4-point Likert scale. The raw sum score of the SDS ranges from 20 to 80 but results are usually presented as the SDS Index, which is obtained by expressing the raw score is converted to 100 points scale [16]. The BDI-21 contains 21 items with a score range of 0–63 that reflects the cognitive, affective, somatic, and vegetative symptoms of depression [15]. We used statistical analyses to determine the optimal cut of point on the SDS score for major depression, then compared the sensitivity and specificity of our cut off point with those of other commonly used threshold values.

Diagnoses of single or recurrent episode of major depressive disorder (major depression) was made according to the DSM-IV criteria [2] using a short structured diagnostic interview, the Mini Neuropsychiatric Interview (M.I.N.I. 5.0.0) [9], by two professional psychiatrists working separately. The psychiatrists were blinded to the results of the above questionnaires while conducting the interview.

### Statistical analyses

We compared the baseline characteristics of the participants with a diagnosis of major depression with those of the subjects without a diagnosis using Pearson’s chi-squared test. We calculated the sensitivity, specificity, and positive and negative likelihood ratios (LR + and LR −), as well as the positive and negative predictive value with 95% confidence intervals (CI) of the SDS for detecting major depression. The SDS cut-off points ≥40, ≥45, and ≥ 50 were chosen based on literature [20,22,23]. To measure the effectiveness of the scale and to select an optimal threshold value for depression diagnosis, we calculated Youden’s index for each cut-off point and generated a receiver operating characteristic (ROC) curve. The ROC-curves were compared using the method described by DeLong and colleagues. The Bayesian Information Criterion (BIC) was used to compare the SDS cut-off points. All statistical analyses were performed using Stata (StataCorp. 2013, Stata Statistical software: Release 13. College Station, TX: StataCorp LP).

## Results

Of the original 667 invited participants, 520 participated into the study. Table 1 presents the general characteristics of the participants. Of the 520 subjects 309 were female. All subjects were 72–73 years old. Major depression defined as a single or recurrent episode according to the DSM-IV criteria, was present in 5.2%. There were statistically significant differences between depressed and non-depressed subjects in terms of their SDS total, positive and negative scores but not their somatic scores.

**Table 1.** Characteristics of the study population (*N* = 520).

		Based on M.I.N.I.		*p*
	Total	Non-depressed	Depressed	
	n	n (%)	n (%)	
Total	520	493 (94.8%)	27 (5.2%)	
Gender				.111
Male	211	204 (96.7%)	7 (3.3%)	
Female	309	289 (93.5%)	20 (6.5%)	
Marital status				.703
Married/Cohabit	343	328 (95.6%)	15 (4.4%)	
Single	24	23 (95.8%)	1 (4.2%)	
Widower/Widow	129	121 (93.8%)	8 (6.2%)	
MMSE, mean (std)	26.6 (2.3)	26.6 (2.3)	26.8 (2.1)	.603
Missing data	7 (1.4%)	5 (1.0%)	2 (7.4%)	.046^a^ exact
Any chronic diseases				.965
No	37	35 (94.6%)	2 (5.4%)	
Yes	420	398 (94.8%)	22 (5.2%)	
Zung’ score				
Total	34.9 (7.7)	34.3 (7.2)	45.6 (8.0)	<.001
Missing data	18 (3.5%)	15 (3.0%)	3 (11.1%)	.060^a^ exact
Negative symptoms	12.9 (2.9)	12.7 (2.6)	17.2 (3.4)	<.001
Positive symptoms	16.7 (5.0)	16.3 (4.8)	22.6 (4.4)	<.001
Somatic symptoms	5.3 (1.6)	5.2 (1.6)	5.8 (1.8)	.104
BDI-21	7.2 (5.7)	6.6 (5.1)	16.6 (6.9)	<.001
Missing data	46 (8.9%)	44 (8.9%)	2 (7.4%)	1.000^a^ exact

BDI-21: 21-item Beck Depression Inventory; M.I.N.I.: Mini Neuropsychiatric Interview; MMSE: Mini-Mental State Examination.

^a^t-test, Chi-Square test or as appropriate Fisher’s Exact test.

Eighteen subjects (3.5%) had incomplete SDS data and 42 (8.9%) provided incomplete BDI-21 questionnaires. Of those 42 subjects with incomplete BDI-21 data, 25 subjects (61%) had not completed the question concerning libido. Table 2 presents the sensitivity, specificity, Youden´s index, and likelihood ratios for the different cut-off points of the SDS. The optimal cut-off point based on Youden index was 39. The sensitivity and specificity parameters for this cut-off point were 79.2% (95% CI 57.8–92.9) and 72.2% (95% CI 67.9–76.1), respectively; its positive predictive value was 12.5% (95% CI 7.7–18.8) and its negative predictive value was 98.6% (95% CI 96.7–99.5). There were no significant differences between the sensitivity and specificity of our optimal cut-off point (39) and those of other commonly used thresholds (40, 45, 50) [20,22,23].

**Table 2.** Screening parameters of the SDS for detecting current major depression based on Mini Neuropsychiatric Interview (M.I.N.I.) results.

	Zung self-rating depression Scale, cut-off points			
	≥39		≥40	
Current major depression	Positive	Negative	Positive	Negative
Yes	19	5	18	6
No	133	345	119	359
Sensitivity	79.2 (57.8–92.9)		75 (53.3–90.2)	
Specificity	72.2 (67.9–76.1)		75.1 (71–78.9)	
AUC	0.76 (0.67–0.84)		0.75 (0.66–0.84)	
Youden's index	0.51		0.50	
Likelihood ratio (+)	2.85 (2.21–3.66)		3.01 (2.28–3.98)	
Likelihood ratio (−)	0.29 (0.13–0.63)		0.33 (0.17–0.67)	
Odds ratio	9.86 (3.73–26)		9.05 (3.61–22.6)	
Positive predictive value	12.5 (7.7–18.8)		13.1 (7.98–20)	
Negative predictive value	98.6 (96.7–99.5)		98.4 (96.5–99.4)	

**Figure 1.**
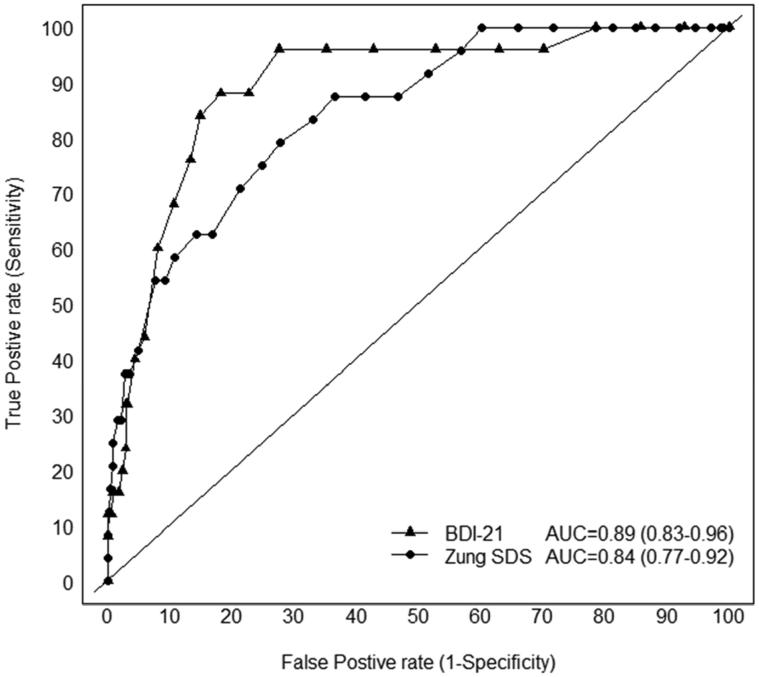
Receiver operating characteristics (ROC) curves for the BDI-21 and SDS.

Figure 1 shows the ROC curve of the SDS (total score) as well as the BDI-21 for detecting the presence of major depression. In the ROC analysis, the area under the curve (AUC) total score was 0.85 (95% CI 0.77–0.92) for the SDS and 0.89 (95% CI 0.83–0.96) for the BDI-21, *p* = .137 (DeLong test). In addition, we calculated area under the curve as 0.82 (95% CI 0.75–0.89) for the SDS positive score and 0.85 (95% 0.78–0.93) for the negative score.

## Discussion

### Statement of principal findings

The aim of the present study was to investigate the psychometric properties of the SDS using the DSM-IV diagnosis of depression as a gold standard, and to compare the screening parameters of the SDS with that of the BDI-21 among the elderly population at the community level. We found that the SDS is a valid screening tool for depression in elderly, with an optimal cut-off point of 39. The results from ROC analyses of our current data, indicated that the AUC did not differ between the SDS and BDI-21. This suggest that the SDS and BDI-21 perform similarly in screening older adults with major depression.

### Strengths and weaknesses of the study

The main strengths of our study are methodological: the homogeneous age of the study group (72–73 years), the use of a psychiatric interview (M.I.N.I.) as the comparator, and the arrangement that the psychiatrists conducting the interviews were blinded to the results of the screening questionnaires.

A limitation of the study is the missing data. With self-administered questionnaires, it is inevitable that some subjects will leave some questions unanswered for one reason or another. Interestingly, a higher proportion of SDS questionnaires (96.5%) than BDI-21 questionnaires (92.1%) were completed. We found that of those with incomplete BDI-21 data, 61% had not answered the question about libido. This may be because these subjects considered it too delicate a question to answer in such a survey. Other studies have also found that the response rate on questions about sexual activity is low [29].

### Findings in relation to other studies

According to the literature, there are different cut-off points used for the SDS [25]. The optimal cut-off point for the SDS among our population was 39. However, it was only slightly more sensitive than the traditionally used cut-off point 40. We found the sensitivities of the other commonly used cut-off points (45 and 50) were significantly lower. Based on our results, these two higher cut-off points may not be meaningful for screening purposes in an elderly population. On the other hand, some authors have reported that the SDS cut-off score should be higher (50 or 60) in medical settings and for elderly subjects [17]. Campo-Arias *et al* validated the SDS among a general population in Colombia (*n* = 266) using the cut-off point 40, however, the sensitivity and specificity of the SDS among his study group was better [24]. On the other hand, the sample was also younger, 18–65 years old [24].

From a clinical standpoint, when administering questionnaires to elderly patients [20,21], shorter forms are more practical. Interestingly, our ROC analysis found that the eight-item ‘negative’ question subset of the SDS performed similarly to the overall 20-item total score. This prompts the question as to whether the negative sub-section alone could be used as a screening tool in the clinical setting. However, since a depression diagnosis does not only consist of negative items, it is important to retain the positive elements of the screening tool.

### Meaning of the study

According to a recent systematic review [30], the validity of screening for depression in the general population is not supported by evidence from randomised, controlled trials. However, in the clinical setting, the use of the screening tools is still important in detecting depression. Therefore, validation studies are required to inform clinical practice. Based on our findings, the SDS is useful in identifying major depression among older adults at the community level. Additional studies are needed further to elucidate the psychometric properties of the SDS.

## Conclusion

While the BDI-21 is one of the most commonly used depression screening scales, we suggest that the SDS is also a convenient tool in identifying clinically meaningful depressive symptoms in older adults. The sensitivity and specificity of these two screening tools are comparable, at least in an elderly Finnish population. Further investigations in other populations are called for.
